# A Presentation of Babesiosis in the Setting of Low-Grade Follicular B-cell Lymphoma

**DOI:** 10.7759/cureus.52585

**Published:** 2024-01-19

**Authors:** Erika Foerst, Karthik Shankar, Jing Zhou, Arezoo Ghaneie

**Affiliations:** 1 Internal Medicine/Pediatrics, MedStar Georgetown University Hospital, Washington, USA; 2 Hematology and Medical Oncology, Lankenau Medical Center, Wynnewood, USA; 3 Laboratory Services and Pathology, Lankenau Medical Center, Wynnewood, USA

**Keywords:** non-hodgkin’s lymphomas, maltese cross, b-symptoms, tick-borne illness, follicular lymphoma, rituximab, ixodes tick, hemolytic anemia, lymphoma, babesiosis

## Abstract

Babesiosis is a tick-borne parasitic infection seen in the Northeast and upper Midwest regions of the United States. Clinically, this intra-erythrocytic parasitic infection can present in a variety of ways, including fever, fatigue, malaise, or myalgia. Of note, these presenting symptoms are very similar to symptoms that can also be seen in patients with low-grade lymphoma. Thus, differentiating between babesiosis infection and active, symptomatic low-grade lymphoma can be difficult. We present a patient with concurrent severe babesiosis infection and follicular lymphoma. This case report provides a unique overlap of Hematology/Oncology and Infectious Disease and the ensuing diagnostic challenges when both tick-borne illnesses and low-grade lymphoma present together. We suggest including babesiosis screening in the pretreatment evaluation for the use of rituximab in patients with the above symptomatology and geography. This will help rule out alternate confounding diagnoses of babesiosis infection before initiating immunosuppressive treatment for active, symptomatic low-grade lymphoma. Using immunosuppressive agents such as rituximab to treat suspected low-grade lymphoma, before ruling out tick-borne illnesses, can be harmful. Our goal is to reduce such instances.

## Introduction

Babesiosis is a tick-borne parasitic disease, primarily transmitted by the species *Babesia microti*. The life cycle of all Babesia species involves internal sporozoite formation [1]. Mature sporozoites are then transmitted from the infected tick to human hosts [1]. These sporozoites enter human red blood cells and replicate asexually, resulting in the known clinical complications as below [1]. Largely seen in the Northeast and upper Midwest regions of the United States, this intra-erythrocytic parasitic infection can present in a variety of ways, mainly in the warmer summer months. The spectrum of disease severity ranges from asymptomatic to a potentially fatal clinical course [2]. Following a one to four-week incubation period after initial infection, common symptoms include fever, fatigue, malaise, and myalgia. Of note, these presenting symptoms are very similar to symptoms that can also be seen in patients with low-grade lymphoma. Hemolytic anemia is commonly seen with babesiosis on laboratory evaluation [2]. Autoimmune hemolytic anemia can be seen in association with low-grade lymphomas, though the incidence is far less common compared to hemolytic anemia seen in babesiosis infection [3]. Severe cases of babesiosis may be seen in patients with underlying malignancy, asplenia, human immunodeficiency virus (HIV) infection, neonatal prematurity, or those on immunosuppressive medications [2].

Co-infection with other tick-borne illnesses, such as Lyme disease or rickettsia can occur, particularly if a dermatologic manifestation is present. Dermatologic manifestations are more common with Lyme disease and rickettsia [4,5]. Erythema migrans can be pathognomonic for Lyme disease [4]. A maculopapular rash around the wrist and ankles is associated with rickettsia [5]. Other differential diagnoses for babesiosis may include malaria, viral hepatitis, bacteremia, non-infectious hemolytic anemia, or HELLP syndrome (hemolysis, elevated liver enzymes, low platelets) [2].

The diagnosis of babesiosis should be suspected when the aforementioned clinical symptoms are seen in a patient with appropriate geographic or epidemiologic exposure. Diagnosis is further confirmed by both laboratory evaluation (polymerase chain reaction test or serology) and blood smear evaluation (characteristic Maltese cross inside red blood cells) [1]. We present a patient with concurrent severe babesiosis infection and follicular lymphoma. This case report provides a unique overlap of Hematology/Oncology and Infectious Disease. We suggest including babesiosis screening in the pretreatment evaluation for the use of rituximab in patients with the above symptomatology and geography, to rule out alternate confounding diagnosis of babesiosis infection, before initiating immunosuppressive treatment for active, symptomatic low-grade lymphoma. Using immunosuppressive agents such as rituximab to treat suspected low-grade lymphoma, before ruling out tick-borne illnesses, can be harmful. Our goal is to reduce such incidences.

## Case presentation

A 60-year-old, Caucasian male living in Pennsylvania presented in the summer with abdominal pain. He stated it was acute in nature, first noticed one week before presentation. The pain was located in the epigastric region. It was episodic and unrelated to food intake. A computed tomography (CT) of the abdomen and pelvis showed diffuse abdominal and pelvic lymphadenopathy. Positron emission tomography-computed tomography (PET-CT) showed multiple enlarged lymph nodes in the retrocrural, inguinal, mesenteric, and retroperitoneal regions. The standardized uptake value (SUV) ranged from 4 to 7.7. For reference, normal background SUV activity of the liver was noted to be 3. Thus, the multiple above-enlarged lymph nodes were deemed avid.

**Figure 1:**
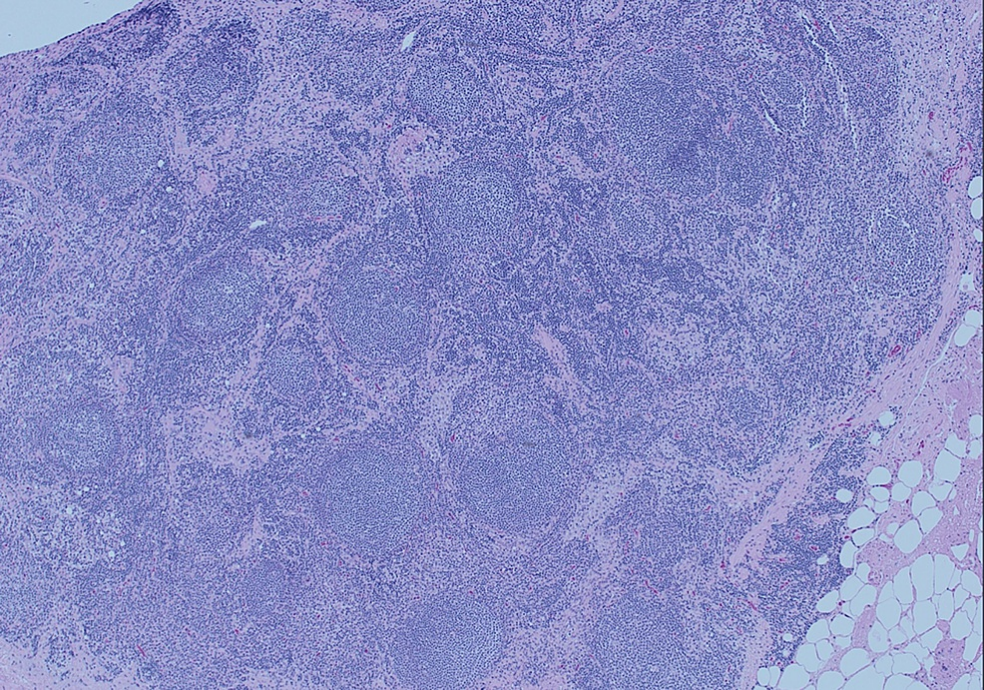
Low power, mesenteric excisional lymph node biopsy.

**Figure 2:**
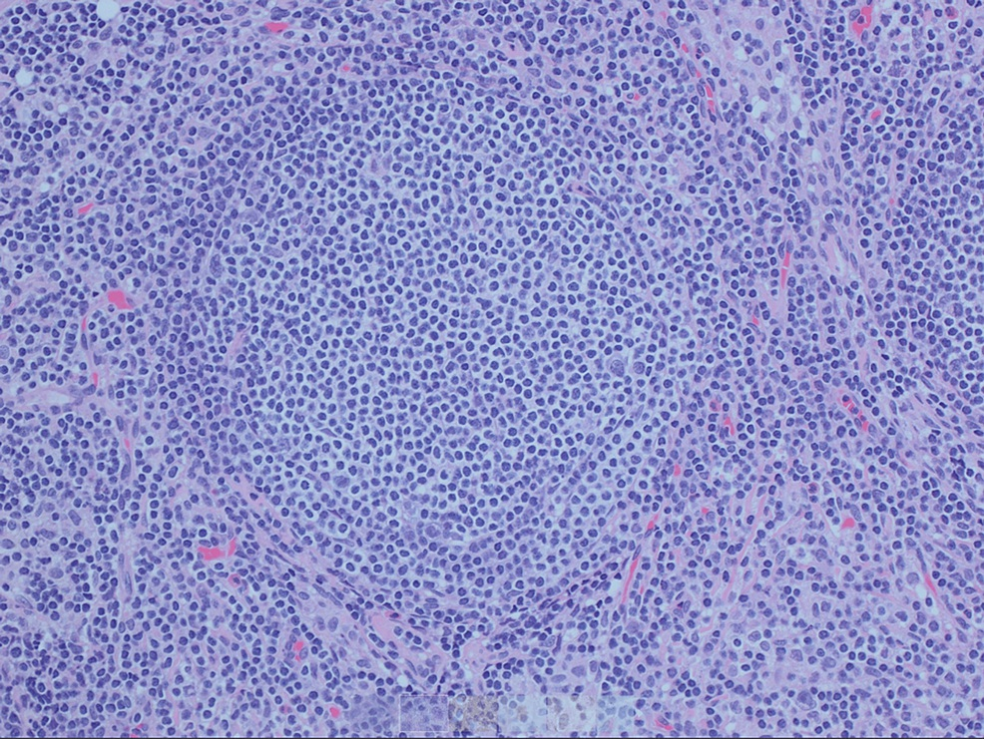
High power, mesenteric excisional lymph node biopsy.

**Figure 3:**
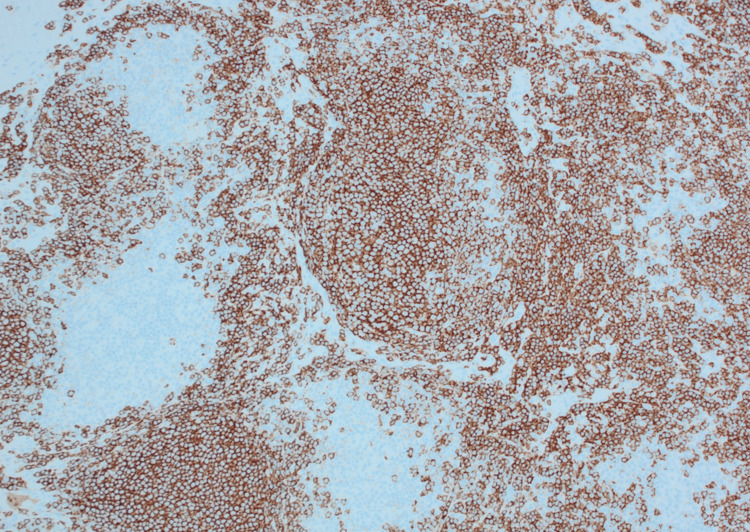
CD20 stain, mesenteric excisional lymph node biopsy.

**Figure 4:**
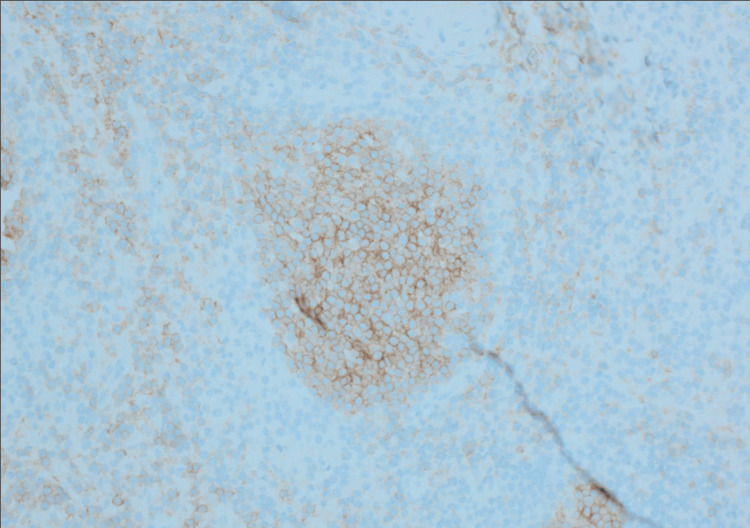
CD10 stain, mesenteric excisional lymph node biopsy.

**Figure 5:**
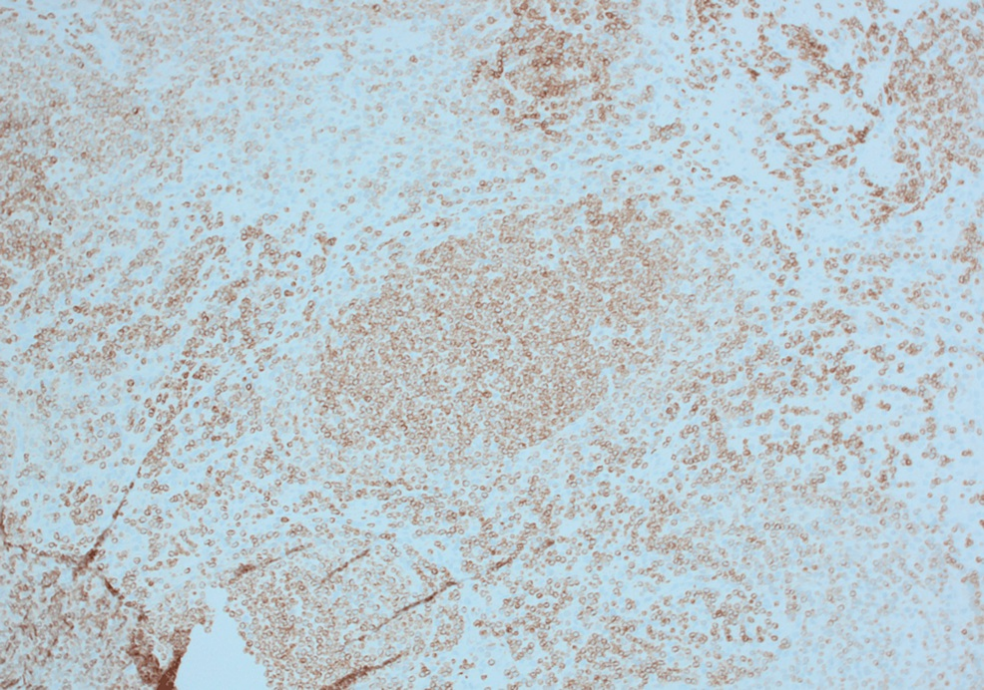
BCL-2 stain, mesenteric excisional lymph node biopsy.

**Figure 6:**
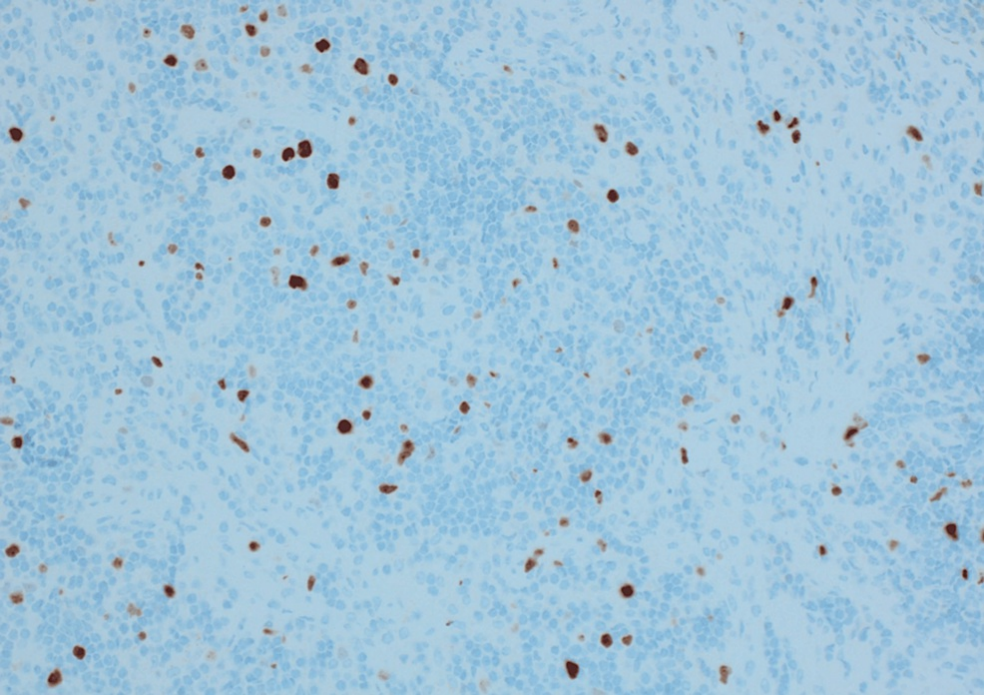
Ki-67, mesenteric excisional lymph node biopsy.

Fine-needle aspiration of a porta hepatis lymph node showed atypical small lymphocytes. Flow cytometry of the tissue showed a population of 60% CD10 positive lambda light chain restricted B cells. Subsequent mesenteric excisional lymph node biopsy showed grade 1 to 2 follicular lymphoma. The diagnosis was supported by immunohistochemical stains (Figures 1-6) of atypical lymphoid cells expressing positive CD20, PAX5, CD10, BCL-6 (weak), and BCL-2. Immunohistochemical stains were negative for CD5 and cyclin D1. It was felt that the acute nature of his abdominal pain was unrelated to the adenopathy noted on the PET scan. Close monitoring was felt to be an acceptable initial management approach.

Approximately three months later, in the fall, the patient started to experience B symptoms with fevers, chills, and night sweats. He also started to experience nausea, bouts of diarrhea, malaise, fatigue, and lethargy. Repeat imaging showed abnormal hypermetabolic lymph nodes predominantly in the retroperitoneum and mesentery. These lymph nodes had enlarged, but there were no signs of lymphoma progression based on the SUV measurements. Three months later, in the winter, the decision was made to start treatment with the regimen of bendamustine and rituximab for what was thought to be symptomatic follicular lymphoma. Before the initiation of treatment, the largest retroperitoneal lymph node was noted to be 3.3 x 2.2 cm, with an SUV of 7. His lactate dehydrogenase was elevated to 355 IU/L before treatment. His blood counts were unremarkable as well with hemoglobin of 13.7 g/dL and platelet count of 154 k/µL. Hepatitis and HIV were ruled out before the initiation of therapy. His immune system, in the absence of other significant medical histories, was felt to be robust.

Four days after completion of his third cycle of bendamustine and rituximab chemotherapy, given along with granulocyte colony-stimulating factor (G-CSF) support, he presented to the hospital with persistent and progressive diarrhea, ultimately leading to excess fatigue. On arrival, he was febrile with a temperature of 102.9°F, blood pressure of 147/74 mmHg, heart rate of 108 beats/minute, respiratory rate of 12 breaths/minute, and oxygen saturation of 97% on room air. His lab work showed mildly elevated total bilirubin to 1.9 mg/dL and mildly elevated creatinine to 1.5 mg/dL. Other notable lab work included elevated white blood cell count of 36.31 k/µL, hemoglobin of 12.3 g/dL, and low platelet count of 101 k/µL. His hemoglobin and platelet count quickly declined to 8.9 g/dL and 58 k/µL, respectively, over the next 48 hours. The hemolysis panel showed elevated lactate dehydrogenase of 358 IU/L, elevated reticulocyte count to 5.96%, and decreased haptoglobin to less than 3 mg/dL. The direct antiglobulin test was negative.

The patient continued to experience fevers throughout his hospital course. He then developed myalgia, headache, and ongoing fatigue. His physical examination was unrevealing. Further review of the patient’s history revealed that he frequently hiked in the woods and mountains in southern Pennsylvania. He reported a tick bite approximately four months before presentation, in the winter months sometime between October and February. He denied any overseas travel or prior blood transfusions. He was up to date with his vaccinations, including coronavirus disease 2019 (COVID-19).

Results

An infectious workup revealed a negative viral panel (which included COVID-19, influenza A/B, and respiratory syncytial virus). A chest X-ray did not show signs of pneumonia, and blood cultures were negative for aerobic and anaerobic organisms. Lyme testing was negative for IgM, but positive for IgG at a titer of 1.70 (reference range of less than 0.90 reflecting a negative test).

A peripheral blood smear was obtained to further evaluate laboratory evidence of hemolytic anemia. The peripheral blood smear revealed intracellular and extracellular ring forms consistent with babesiosis. A picture of the peripheral blood smear was requested but was unable to be obtained. Parasitemia level was 4.5%. Subsequent polymerase chain reaction (PCR) blood test was positive for *Babesia microti *DNA. Thus, a diagnosis of babesiosis infection was made.

The patient was treated for babesiosis infection with intravenous (IV) azithromycin and oral atovaquone. His diarrhea, fatigue, lethargy, nausea, fevers, and chills resolved. He completed a six-week course of treatment. Parasitemia levels had normalized for two weeks before both medications were stopped in the outpatient setting.

He was also treated with a 14-day course of doxycycline due to the positive Lyme antibodies. Chemotherapy for the treatment of his follicular lymphoma was temporarily held throughout the entire duration of his treatment for babesiosis and Lyme infections.

Two weeks after completion of antibiotic therapy for babesiosis infection, a repeat PET scan was done in March 2022 which showed complete resolution of his abdominal and pelvic lymphadenopathy. No metabolically active lymph nodes were noted. He has been monitored off anti-neoplastic therapy since then.

## Discussion

On further review of the literature, there have been prior case reports that present a *Babesia microti* infection in the setting of a malignant hematologic process. Specifically, Conte et al. described babesiosis in a 76-year-old male who was previously diagnosed with indolent chronic lymphocytic leukemia [6]. The patient originally presented with anemia, pancytopenia, and splenomegaly, which was presumed to be a malignant transformation of the patient’s known hematologic malignancy, for which he was given antioxidant supplementation [6]. However, despite subsequent treatment for the initial suspected diagnosis, he continued to clinically decline. Finally, ring-form intra-erythrocytic parasites were identified on a peripheral blood smear, and an rt-PCR blood test confirmed *Babesia microti* DNA in the blood, which confirmed the diagnosis of babesiosis infection [6]. He was treated with atovaquone and azithromycin, leading to recovery [6]. This patient subsequently had two recurrences of the babesiosis infection thereafter [6].

Furthermore, although more commonly seen in North America, there was also an account of babesiosis in a 63-year-old, splenectomized male in southern Germany [7]. Four weeks after being treated with rituximab for an unspecified B-cell lymphoma, the patient presented with dark urine, jaundice, and labs consistent with hemolytic anemia and renal insufficiency [7]. Blood smears revealed *Plasmodium*-like intra-erythrocytic organisms; however, PCR confirmed the presence of babesiosis EU1 [7]. The patient was originally treated with quinine and clindamycin, which led to his initial remission, but then required atovaquone and azithromycin due to relapse of his condition [7]. Additional reports of babesiosis infection in malignant hematologic patients include babesiosis secondary to a red blood cell transfusion in a patient with refractory lymphoma [8].

Other parasitic infections, such as rickettsia (which are also transmitted via infected tick vector bite into human hosts) were also diagnosed in patients with hematologic malignancy. For example, a patient with non-Hodgkin’s lymphoma was found to have boutonneuse fever, which is caused by rickettsia [9]. This clinical course was complicated by significant pulmonary involvement, but the patient was treated with doxycycline, leading to the resolution of his infection [9].

Both our case report, as well as the other literary sources described above, illustrate perspectives of the life-threatening clinical courses of babesiosis that can develop in patients who are immunocompromised, specifically in the setting of a hematologic malignancy. Our case provides additional insights into the literature on the unique association between babesiosis infection and lymphoma. Our case highlights the importance of considering babesiosis in the differential diagnosis of an immunocompromised patient, especially with underlying hematologic malignancy. Without prompt recognition of this association, babesiosis infection can cause rapid and potentially fatal clinical deterioration. In fact, if babesiosis is not considered, treating low-grade lymphomas with immunosuppressive agents, such as rituximab in our case, may accelerate the clinical deterioration seen from tick-borne illnesses. Immunosuppressive agents may also increase the severity of symptoms caused by tick-borne illnesses, leading to other unexpected clinical complications.

Of note, when further analyzing our specific case, it is essential to recognize that the presenting symptoms of low-grade lymphoma and babesiosis, including B-symptoms, fever, chills, anorexia, hemolytic anemia, and splenomegaly, have significant overlap. Although asymptomatic low-grade lymphoma is typically managed conservatively with initial observation [10], these non-specific flu-like symptoms seen in our patient led to his treatment with bendamustine and rituximab chemotherapy, along with G-CSF support. Had his babesiosis infection been identified at the time of his presentation, treatment of his follicular lymphoma would not have been necessary. Rather, the rituximab caused the patient to have increased immunosuppression, leading to his hospitalization and more severe clinical decompensation from underlying and masked babesiosis. Although screening for infections, such as HIV, tuberculosis, and hepatitis B/C, is routinely done before treating a patient with rituximab, one could consider modifying this practice to include screening for babesiosis in patients with the above symptomatology, especially in those living in endemic areas [11].

Lastly, our case report highlights an important aspect in the diagnosis of babesiosis, i.e., the effect of global warming and climate change on both the diagnosis time and space of *Ixodes *tick-borne illnesses. Most cases of babesiosis, in endemic regions of the Northeastern United States (Pennsylvania, Massachusetts, Rhode Island, Connecticut, and New York) occur between the summer months of July through August [12]. With the progression of climate change over the last 20 years, *Ixodes *tick populations have been able to survive in northern regions that were previously cooler for most of the year and not conducive to *Ixodes *tick survival [13]. Thus, it can be inferred that the endemic region of *Ixodes *tick-borne infections will expand into the Canadian states of Ontario and Quebec in the near future [13]. Furthermore, it can be inferred that *Ixodes *tick-borne infections will occur more frequently out of season [13]. In our patient, this was the case, as a diagnosis of babesiosis was made between the months of October and February.

Upon a review of the literature, there has already been evidence of increased incidence of tick-borne illness in more northern regions of Canada [14]. Between the years of 2009 to 2015, a Canadian surveillance study showed that Lyme disease was on the rise [15]. There was a six-fold increase in the number of reported Lyme disease cases from 2009 to 2015 [15]. Furthermore, the geographic region of Canada where *Ixodes *ticks were identified significantly expanded from 2009 to 2015 [15].

With regards to babesiosis specifically, a Canadian study was done in 2016 to assess the prevalence of *Babesia microti* infection in Canadian territories. The study was conducted by examination of 12,000 ticks across different provinces of Canada [16]. Results showed that *Babesia microti* infection was identified in ticks from four different Canadian provinces (Manitoba, Ontario, Québec, and New Brunswick) [16]. At the time of the study, human babesiosis infection had only been identified in Manitoba [16]. Thus, the study inferred that *Babesia microti*-infected ticks could be advancing into different parts of Canada, where they previously had not been encountered [16]. This could ultimately lead to a higher incidence of human babesiosis infections in these regions in the near future.

## Conclusions

Overall, we feel that our case report adds to the literature in a variety of ways. It helps keep tick-borne illnesses on our differential when evaluating a patient with lymphoma. It also helps illustrate the continued impact climate change can have on the incidence of future tick-borne illnesses, both in number and in geographical territory. Thus, we suggest the addition of pretreatment babesiosis screening before the use of immunosuppressive therapies such as rituximab in endemic regions. Continued study of tick-borne illnesses in association with lymphoma can help enhance our knowledge in both of these aspects.
